# A Flexible Approach for Highly Multiplexed Candidate Gene Targeted Resequencing

**DOI:** 10.1371/journal.pone.0021088

**Published:** 2011-06-30

**Authors:** Georges Natsoulis, John M. Bell, Hua Xu, Jason D. Buenrostro, Heather Ordonez, Susan Grimes, Daniel Newburger, Michael Jensen, Jacob M. Zahn, Nancy Zhang, Hanlee P. Ji

**Affiliations:** 1 Division of Oncology, Department of Medicine, Stanford University School of Medicine, Stanford, California, United States of America; 2 Stanford Genome Technology Center, Stanford University, Palo Alto, California, United States of America; 3 Biomedical Informatics Program, Stanford University, Stanford, California, United States of America; 4 Department of Statistics, Stanford University, Stanford, California, United States of America; Smithsonian Institution National Zoological Park, United States of America

## Abstract

We have developed an integrated strategy for targeted resequencing and analysis of gene subsets from the human exome for variants. Our capture technology is geared towards resequencing gene subsets substantially larger than can be done efficiently with simplex or multiplex PCR but smaller in scale than exome sequencing. We describe all the steps from the initial capture assay to single nucleotide variant (SNV) discovery. The capture methodology uses in-solution 80-mer oligonucleotides. To provide optimal flexibility in choosing human gene targets, we designed an *in silico* set of oligonucleotides, the Human OligoExome, that covers the gene exons annotated by the Consensus Coding Sequencing Project (CCDS). This resource is openly available as an Internet accessible database where one can download capture oligonucleotides sequences for any CCDS gene and design custom capture assays. Using this resource, we demonstrated the flexibility of this assay by custom designing capture assays ranging from 10 to over 100 gene targets with total capture sizes from over 100 Kilobases to nearly one Megabase. We established a method to reduce capture variability and incorporated indexing schemes to increase sample throughput. Our approach has multiple applications that include but are not limited to population targeted resequencing studies of specific gene subsets, validation of variants discovered in whole genome sequencing surveys and possible diagnostic analysis of disease gene subsets. We also present a cost analysis demonstrating its cost-effectiveness for large population studies.

## Introduction

Next generation DNA sequencers have substantially expanded our ability to survey human genomes for germline variants or the somatically acquired mutations characteristic of cancer [1], [2], [3], [4], [5], [6]. For many research studies and applications, targeted resequencing of specific genomic regions such as candidate genes, is a generally useful approach for validating mutations in newly discovered disease genes, detecting rare variants from populations and screening for polymorphisms of interest. While exome sequencing has become commonly available, there are numerous applications and studies which oftentimes only require a significantly smaller scale of targeted resequencing. Examples of informative subsets of genes include mutation analysis of Mendelian disorder genes with extensive genetic heterogeneity. For example, there are over forty causative genes for hypertrophic cardiomyopathy but clinical testing is restricted to only a small number of these genes for a given clinical analysis [7]. Another application is for the follow up validation of somatic mutations in cancer genes sets identified from exome surveys of large collections of tumors [8], [9]. Frequently there is a requirement for additional validation of mutations from subsets of candidate cancer genes to confirm that mutations are not simply passenger mutations with no significant biological or clinical significance. Genome wide association studies involving thousands of individuals are increasingly being geared towards resequencing specific loci for the identification of rare variants and would be facilitated by high throughput approaches for capturing specific loci [10]. For the validation of large number of variants identified in complete genome or exome surveys, validation targeted resequencing using next generation platforms has proven to be an attractive alternative to Sanger sequencing.

For those researchers seeking to analyze candidate gene sets for mutations, polymorphisms and other variants with next generation sequencers, we have developed a robust approach using in-solution capture mediated by pools of 80-mer oligonucleotides. Our capture technology is geared towards resequencing gene subsets substantially larger than can be done efficiently with simplex or multiplex PCR but reduced in scale compared to exome sequencing. This method is highly flexible as nearly any gene can be targeted and the assay can be implemented with standard molecular biology infrastructure in a short period of time. Nanogram amounts of starting genomic DNA are all that is required which is significantly less than is required for commercially available capture assays [11], [12]. Our method has been successfully applied to targets ranging in size from 100 Kilobases (Kb) to a Megabase (Mb). We also demonstrated the application of sequence indexing to increase sample throughput.

To provide optimal flexibility in capturing human gene targets, we designed an *in silico* set of oligonucleotides that capture the gene exons annotated by the Consensus Coding Sequence (CCDS) Project [13]. We refer to this resource as the Human OligoExome which is publically available via a website (oligoexome.stanford.edu). Using oligonucleotide sequences derived for the Human OligoExome, this approach allows researchers to design capture assays using any arbitrary set of CCDS annotated exons and subsequently resequence them in highly multiplexed capture reactions. The *in silico* set has quality control features to improve the performance of capturing exons from the CCDS reference set.

This in-solution method uses selective genomic circularization to capture specific restriction fragments (Fig. S1) [14]. This method differs from the molecular inversion probe (MIP) approach in that the genomic DNA target is directly incorporated into a circular molecule whereas in MIP technology, the oligonucleotide is converted to an intact circle through polymerase extension using the genomic DNA target as a template [15]. The capture reaction requires a restriction enzyme digest of nanogram amounts of genomic DNA followed by incubation with a mixture of two types of oligonucleotides: i) pools of capture oligonucleotides which are specific for a targeted genomic region and ii) a general vector oligonucleotide. Each capture oligonucleotide is an 80-mer and has two single-stranded target complementary end-sequences (20 nucleotides each) that are linked by a general sequence motif (40 nucleotides). For each oligonucleotide, the complementary flank sequences, referred to as capture arms, mediate the selective circularization of the genomic DNA target, which has been cut by a specific restriction enzyme. The universal vector oligonucleotide is complementary to the general sequence motif in every targeting oligonucleotide. After annealing and ligation, the vector sequence is incorporated into the selected genomic circles. The vector sequence contains a universal primer-pair that mediates the amplification of all of the targeted genomic contents of the circle. A next generation sequencer such as the Illumina Genome Analyzer is used to interrogate the targeted amplicons.

To demonstrate that this approach can accurately identify polymorphisms, variants and mutations in targeted regions of the human genome, we developed several capture assays. We conducted a targeted resequencing analysis of large numbers of exons from normal genomic DNA of individuals in the Hapmap study [16] as well as genomic DNA from a matched normal-tumor pair. For resequencing of the captured genomic DNA, we used an Illumina Genome Analyzer. Relying on the Human OligoExome Resource we designed three different assays that captured the exons of 10 genes (102.48 Kb total), 96 genes (822.15 Kb total) and 106 genes (943 Kb total); the last of these included all of the 96 genes from the second assay. We optimized the assay to accept starting amounts of genomic DNA under 100 ng. We assessed the performance of the assay and developed an adjustment scheme to normalize fold-coverage of captured regions. We successfully integrated indexing to increase sample throughput post-capture. We also provide a cost assessment of our capture assay per sample.

## Results

### Designing capture oligonucleotides for the CCDS exon set

We designed an automated bioinformatic pipeline that relies on *in silico* assessment of different restriction enzymes to create capture oligonucleotides covering the exons defined by the CCDS project. We targeted genes as defined by a specific region-of-interest (ROI), which encompasses exon bases and 50 bases of adjacent intronic sequence. As our first step in developing this process, we evaluated the ability of restriction enzymes to produce intact ROIs. Our initial test set was 244 ROIs from 23 genes. With respect to this method, we ranked the effectiveness of 14 commercially available restriction enzymes recognizing 12 out of the 16 possible 4-base recognition sites (Text S1). Restriction enzymes *Mse*I, *Bfa*I, *Sau*IIIA and *Cvi*QI had the highest design coverage, with greater than 95% of the 244 ROIs covered. We confirmed that these four enzymes ranked highest in terms of providing optimal design coverage by testing a second, separate set of 170 ROIs derived from 10 other genes.

Using the four enzymes that provided optimal coverage (*Mse*I, *Bfa*I, *Sau*3AI and *Cvi*QI) we empirically determined that fragments of up to 800 bases were efficiently captured. The assay we previously described targeted 250 base fragments [14]. This enabled us to design fewer oligonucleotides to cover any given portion of the targeted genome compared to our previous effort. Our results are summarized in Table 1. We used 17,049 genes listed in the CCDS database [13]. The database contains exon definitions for 16,952 genes. The remaining 97 were under review at the time of download with no exon definitions. Exon definitions for these remaining genes came from Genbank. From this set we extrapolated 157,624 ROIs which were then submitted to the TargetedOligoDesign program. The availability of restriction sites and the avoidance of known SNPs appearing in the capture arms were the main design constraints. A total of 784,783 capture oligonucleotides were generated *in silico* in approximately equal proportions for the four restriction enzymes. We designed capture oligonucleotides with substantial redundancy to increase the likelihood that at least one oligonucleotide would capture the target. These four restriction enzymes provided enough sites to adequately design capture oligonucleotides which cover over 98% of ROI bases over all CCDS exons.

**Table 1:** OligoExome design summary.

Parameter	HumanOligoExome
**Total number of genes from CCDS**	17,049
**Total number of ROI sequences**	157,624
**Average ROIs per gene**	9.25
**Total ROI bases derived from CCDS (Mb)**	44.6
**Total number of designed capture oligonucleotides**	784,783
**Number of captured ROI bases by design for all restriction enzymes**	98.3%
**Average number of capture oligonucleotides per gene**	46
**Average number of capture oligonucleotides per ROI**	5

Region-of-interest (ROI) is defined as a minimum of the exon and adjacent intronic sequence up to 50 bases from the exon flank.

We analyzed the sequences of all oligonucleotides' capture arms in order to identify potential issues that might cause failures or off-target capture. Four different quality control factors were reviewed for each oligonucleotide which included the presence of sequences repeated over the human genome (W-flag), paralogs (P-flag), matches to consensus repeats (R-flag) and Alu sequences (A-flag). We identified a total of 97,120 oligonucleotides with at least one flag (Table 2). Each enzyme based set contains a relatively similar number of flagged oligonucleotides. The genome W-flag is the least restrictive and applies to 96,565 oligonucleotides. We verified that all of the P-flag oligonucleotides also had a W-flag. More than 90% of the A-flag and R-flag oligonucleotides also had a W-flag. The small number of differences is attributable to the shorter 14 base sequence used for comparisons in the case of the A and R flags. We examined the reduction in coverage using the quality control flags (Table 2). Excluding the flagged oligonucleotides for all four enzymes provided an average coverage of 94.2% for any given CCDS annotated gene.

**Table 2:** Oligoexome design and flag summary.

Restriction enzyme	*Bfa*I	*Cvi*QI	*Mse*I	*Sau*3AI	Total per category
**Total designed capture oligonucleotides**	190,900	191,315	186,011	216,557	784,783
**Oligonucleotides with no flag**	169,776	171,320	161,813	184,754	687,663
**Oligonucleotides - whole genome** **W-flag**	20,992	19,882	24,021	31,670	96,565
**Oligonucleotides – paralog** **P-flag**	4,915	4,983	4,854	6,179	20,931
**Oligonucleotides – Alu** **A-flag**	290	183	58	460	991
**Oligonucleotides - repeat sequence** **R-flag**	379	379	578	456	1,792
**Total flagged capture oligonucleotides per restriction enzyme (union of all flagged oligos)**	21,124	19,995	24,198	31,803	97,120

### Access to the Stanford Human OligoExome

We deposited all 784,783 capture oligonucleotide sequences into a publically-accessible web-based database application (oligoexome.stanford,edu). Upon arriving at the Human OligoExome website, new users are initially prompted to sign-up for access. Subsequently, users will enter their username and password to access the database. Capture oligonucleotide sequences for specific genes may be retrieved either by entering one or more genes of interest in HUGO symbol format, or by electing to download the set of sequences for the entire exome in a compressed format. The very small number of genes which are not covered are also listed at the Human OligoExome website.

### Designing custom capture assays

We designed three separate capture assays using three restriction enzymes for our initial test and these assays are described in Table 3. We initially selected ten cancer genes (Text S1) where the ROI bases (combined exons and adjacent 50 bp of intronic sequence) covered a total 48.141 Kb of sequence. This smaller target size was useful for optimizing the assay's molecular performance. We designed and synthesized 360 oligonucleotides using the restriction enzymes *Mse*I, *Bfa*I and *Sau*3AI. This 10-gene assay theoretically captures 47.554 Kb of ROI bases (98% of the total) and 54.934 Kb of additional intronic sequence extending further out from the exons for a total capture size of 102.488 Kb. Additional intronic sequences are captured because some capture oligonucleotides utilize a restriction site that exists outside of the 50 bases of intronic sequence adjacent to exons. This additional capture is particularly useful in assessing other genomic regions such as promoters.

**Table 3:** Description of assays.

Genomic Target Specifications	Capture assay 1	Capture assay 2	Capture assay 3
**Total genes**	10	96	106
**Total exons**	179	1776	2021
**Total exon bases (Kb)**	30.446	325.303	362.309
**ROI bases: Total exon plus adjacent 50 bases of intron sequence (Kb)**	48.141	501.489	562.974
**Capture Assay Specifications**			
**Total capture oligonucleotides**	360	4,211	4,792
**ROI bases: Total exon plus adjacent 50 bases of intron sequence (Kb)**	47.554	454.166	512.556
**Intron bases not immediately adjacent to exons (Kb)**	54.934	367.984	430.453
**Total theoretical captured bases (Kb)**	102.488	822.15	943.009
**Percentage covered genomic targets**	98.8%	90.6%	91.0%
**Percentage missed genomic target**	1.0%	9.4%	9.0%
**Annotation of captured region**			
**Repeat masking over exon plus 50 adjacent intron bases (Kb)**	0.468	18.078	19.684

To test larger capture assays, we chose 106 cancer genes (capture assay 3) derived from two previously identified cancer gene sets (Text S1). These genes included the top ranked COSMIC cancer genes as determined by mutation frequency for colorectal and pancreatic cancer [17]. We also included the top ranked cancer genes identified for colorectal cancer [8] and pancreatic cancer [9] from an exome survey of these primary tumors. This target had a ROI size (exon and adjacent 50b) of 562.974 Kb (Table 3). We extracted 4,792 targeting oligonucleotides from the Human OligoExome resource covering these genes and synthesized these oligonucleotides. This assay relied on separate *Mse*I, *Bfa*I and *Cvi*QI reactions. Including all intronic sequences, capture assay 3 theoretically targets 943 Kb. The design captures 512.556 Kb or 91% of the intended ROI bases. We also report some intermediate results based on a 96-gene assay (capture assay 2). Its genes comprise a proper subset of the 106 genes targeted in capture assay 3.

In assessing the theoretical capture size of each assay we noted the lower yield for capture assay 3 (91%) relative to capture assay 1 (98.8%). This is due to the presence of several genes with close homologs in the genome such as *PTEN* which has a well known pseudogene. Capture oligonucleotides for these repetitive gene sequences tend to be paralog flagged in the Human OligoExome resource and while their elimination improves target specificity, it can reduce target coverage. Using improved design algorithms (data not shown) it is often possible to place the targeting arms outside of regions of high homology and thus improve the capture yield for such regions.

### Assessment of targeting performance after capture adjustment

In the initial performance testing of the targeting assay, we created three separate subpools of targeting oligonucleotides in equimolar ratios where each oligonucleotide was at a final concentration of 50 pM in the circularization reaction. Each subpool was specific for a single restriction enzyme. Normal genomic DNA (NA06995 or NA07037) was used in the assessment of the targeting assay. Resequencing was done with an Illumina GAI or GAII using single short read sequences ranging from 36 to 42 bases. To assess the individual success of each capture oligonucleotide, the three subpools were sequenced separately. This prevented overlap among amplicons from different restriction enzymes, allowing us to assess the success of each capture oligonucleotide individually. We aligned the resulting sequence data for each subpool against the reference sequence and determined the average fold-coverage (FC) for each amplicon represented in the subpools.

**Figure 1:**
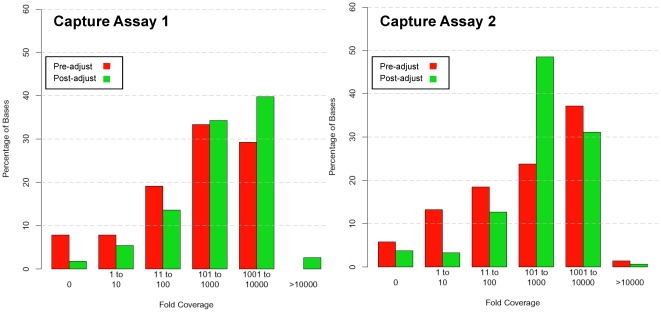
Adjustment of capture oligonucleotides performance. Pre- and post-adjustment capture oligonucleotides performance of capture assays 1 and 2 are shown. Capture assay 1's target size was 102.48 Kb and this intermediate version of capture assay 2 covered 616 Kb. The Y axis shows the proportions of bases across the target whose fold-coverage can be sorted into each order of magnitude before and after capture adjustment. Nominally, we opted for a sequencing depth between 100 and 1,000 as an adequate representation. In both assays, the proportion of bases whose FC is less than 100 drops significantly; in the case of capture assay 2, the number of bases with excessively high FC has dropped significantly as well.

Similar to other approaches used in capturing genomic targets, there is variable representation of any given captured sequence. This variation is attributable to the capture efficiency and the intrinsic properties of the target genomic sequence. To attain a more uniform distribution among the targeted genomic regions, we developed an adjustment scheme. Each oligonucleotide is categorized into one of three performance groups: i) high yield resulting in an average FC greater than 1,000, ii) medium yield resulting in average FC between 100 and 1,000 and iii) low yield resulting in average FC less than 100. For the adjusted capture subpools, the low yielding oligonucleotides' concentration was increased ten-fold while the high yielding were decreased ten-fold. The final concentration of each oligonucleotide in the circularization reaction is 5 pM, 50 pM, and 500 pM for the high yield, medium yield and the low yield groups, respectively. We initially tested this adjustment scheme on capture assays 1 and 2. After concentration adjustment, we observed a decrease in the number of bases with low fold coverage (<100) in both capture assay 1 and 2 (Fig. 1). Specifically for capture assay 2, we also observe a decrease in the number of bases with high coverage (>1000) thus narrowing the coverage distribution. Overall, the variation in the FC distribution decreased compared to the equimolar pools and capture failures decreased significantly. For example, in capture assay 2, the effect of the concentration adjustment include a four-fold decrease in the number of bases with low representation (1 to 10 average FC) and a two fold increase in the number of medium yield (101 to 1,000 average FC) bases.

### Assessing capture performance and specificity

A summary of the perfomance of the three assays is presented in Table 4. The assay was extremely reproducible as demonstrated in two separate replicates of the 106-gene capture assay on NA18507 which was conducted with 50 ng (replicate 1) and 80 ng (replicate 2). Because the design is centered on exons we achieve the highest coverage on exon bases. For example, in capture assay 3 we show that the median FC over all targeted bases is 367. However, if one examines only the exon bases, the median FC is 736. Across all assays, the percentage of all bases with FC above the half median is 60–65%. We also analyzed the capture specificity of the assay. We define nonspecific capture as the percentage of all sequences which do not map to the regions targeted by the capture oligonucleotides but do align to regions of the human genome outside of the targets. For the capture assay 3, the average non-specific capture was 4.6%.

**Table 4:** Variant description and assay performance summary.

Genomic targets	Capture assay 1			Capture assay 2	Capture assay 3	
**Total genes**	10	10	10	96	106	106
					Replicate 1	Replicate 2
**Assay yield**						
**Sample**	NA07037 (CEU)	NA07435 (CEU)	NA06995 (CEU)	NA07037 (CEU)	NA18507 (YRI)	NA18507 (YRI)
**Mapped sequence (Mb)**	90.007	40.907	87.770	1550.928	1279.549	1081.001
**Percentage not captured for assay**	3.6%	4.4%	4.0%	7.3%	9.1%	9.1%
**Percent above ½ median fold coverage**	63%	64%	64%	62%	60%	60%
**Percentage capture coverage 10 or greater**	89.4%	85.4%	89.9%	86.7%	85.0%	84.7%
**Median fold-coverage for assay**	380	151	348	446	367	304
**Hapmap SNP comparison**						
**Heterozygotes (resequencing/array)**	18/18 (100.0%)	13/14 (92.9%)	16/16 (100.0%)	135/137 (98.5%)	160/171 (93.5%)	155/168 (92.2%)
**Homozygotes (resequencing/array)**	42/42 (100.0%)	45/45 (100.0%)	46/46 (100.0%)	344/345 (99.7%)	932/946 (98.5%)	924/936 (98.7%)
**Odds ratio**	Infinite	5,318	Infinite	20,104	952	903
**Other SNVs annotated in dbSNPs**						
**Heterozygotes**	13	11	9	156	162	160
**Homozygotes**	10	12	11	84	59	58

One aspect of our reported results involves the substantial increase in sequence data across time. During the course of our study, the total number of mapped bases increased from 40 to 90 Mb in capture assay 1 to more than one Gigabase (Gb) in both capture assays 2 and 3. The increase in sequence was due to a combination of improved capture, sequencer hardware improvements (GAI to GAII) and image analysis, base calling, and alignment software upgrades (SCS 2.2/Illumina Pipeline 1.01 to SCS 2.6/Pipeline 1.6). As a result of these changes, the proportion of the target over which we make high confidence SNV calls remains relatively constant although the target size increases nearly ten-fold.

To correlate the capture performance as assessed through average FC of a specific oligonucleotide with its individual physical characteristics, we analyzed the post-adjustment performance of the targeting oligonucleotides of capture assays 1 and 2. Our analysis of the average FC for individual oligonucleotides revealed that i) the amplicon length, ii) GC% content of the targeting arm flanks and iii) the flap size of overhanging genomic DNA sequence influenced the performance of the assays (Text S1). For example, in capture assay 1, 24 amplicons were larger than 800 bases. They had an average FC ten times lower than that of the 224 oligonucleotides targeting regions of 200 to 600 bases in size. We observed a similar trend with capture assay 2. High GC content of an individual oligonucleotide's sequence specific capture arms also contributed to poor performance regardless of the genomic capture size of the individual assay. Oligonucleotides with capture arm GC content less than 75% had an average FC two times greater than those oligonucleotides whose capture arms had GC content greater than 75%. The presence of the overhang genomic flap region also contributed to decreased performance. We observed a two-fold drop in average FC for oligonucleotides producing short flaps and a three-fold drop for those producing longer flaps. In optimizing capture assay 3, these observed performance biases were significantly reduced (data not shown).

**Figure 2:**
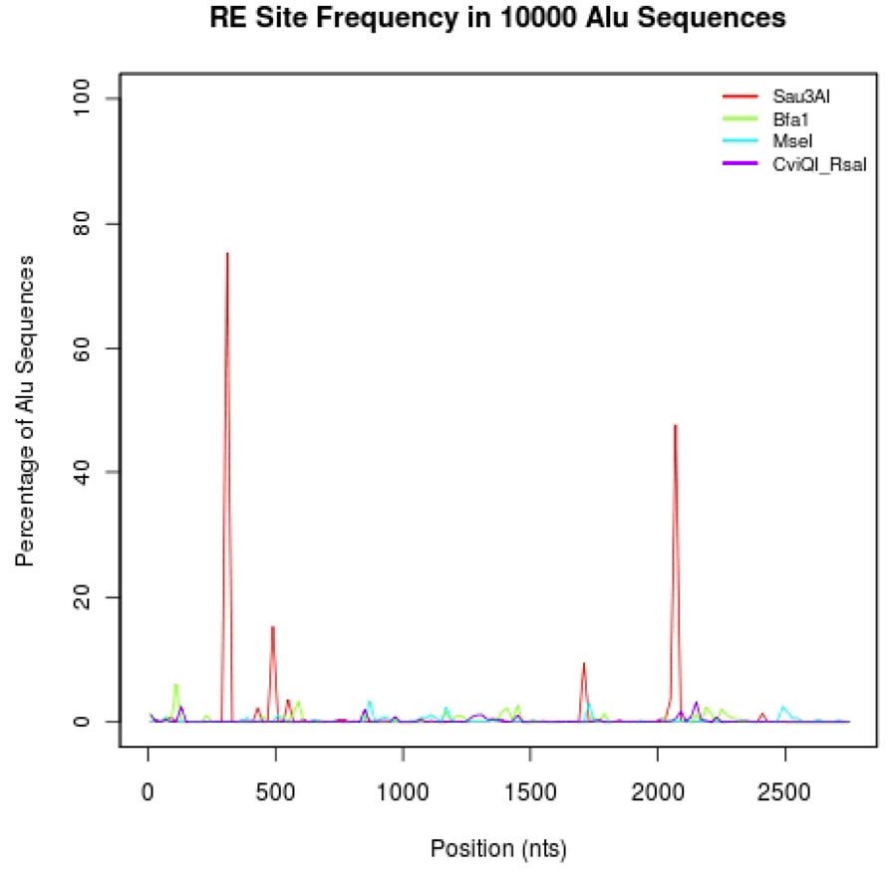
Evaluation of Alu sequence in non-specific capture. Ten-thousand consensus length (297 bases) Alu sequences were randomly selected and aligned. The percentage of Alu sequences containing *Mse*I, *Bfa*I, *Sau*IIIA and *Cvi*QI sites along the multiple alignment positions is shown. The four most prevalent restriction sites are *Sau*IIIA sites. The two most frequent amongst these are present in 50 to 75% of the Alu sequences. We note that the alignment sequence is much longer than many individual Alu sequences because of insertions and deletions.

Analysis of the targeted sequencing results also showed that the *Sau3*AI-based capture oligonucleotides had more off-target capture and amplification than did those derived from the other three enzymes. The number of oligonucleotides with W-, P- and A-flags was higher in the *Sau3AI* set. In particular, we discovered that the consensus Alu sequences contain no *Mse*I, *Bfa*I or *Cvi*QI sites but two *Sau*3AI sites are present. We verified (Fig. 2) that these two sites are highly conserved amongst 10,000 consensus length Alu sequences randomly chosen from the human genome aligned to each other using MUSCLE v. 3.6 (http://www.drive5.com/muscle) [18], [19]. Thus, when the *Sau*3AI capture oligonucleotides are used, a large proportion of these Alu restriction fragments can circularize because of the internal placement of the restriction sites.

### Multiplexing and reproducibility

To investigate the sample-to-sample variability of the entire capture and targeted resequencing process, we analyzed the results from three separate 10-gene assays sequenced in separate sequencing lanes or in multiplexed format with multiple samples per lane. Multiplexed resequencing of samples relies on indexing and is particularly useful for increasing the number of samples in a given sequencing run when high FC is available. We used a four-plex indexing methodology in which the tag is introduced via the Illumina sequencing adapters and the tag consists of a single nucleotide barcode present immediately after the sequencing primer. After the base calling process and alignment, the mapped sequence reads are separately binned based on the barcode. Using single reads and 1-base barcodes (allowing 4-plex indexing) the false assignment of barcode sequence (e.g. “incorrect index assignment” rate) is approximately equal to the sequencing error rate per base (0.5–1% in these samples). We determined that an incorrect index assignment rate of up to 1% does not pose significant problems in terms of accurate genotyping. In the Hapmap genotype comparison we present below, for the true positive heterozygote cases we call, the second allele base percentage is greater than 13%. Below that number, our quality controls metrics eliminate these potential variant calling errors. If a sample containing a homozygote variant is incorrectly indexed and assigned to a different sample, the introduction of the false homozygote will only contribute 1% of the reads to the other sample. As an additional error control, we used barcode indexing on both ends of a mate pair sequences. This dual indexing strategy reduces the incorrect indexing assignment to 0.1%. This low indexing error rate is important in certain applications such as identification of quasi species in viral populations.

**Figure 3:**
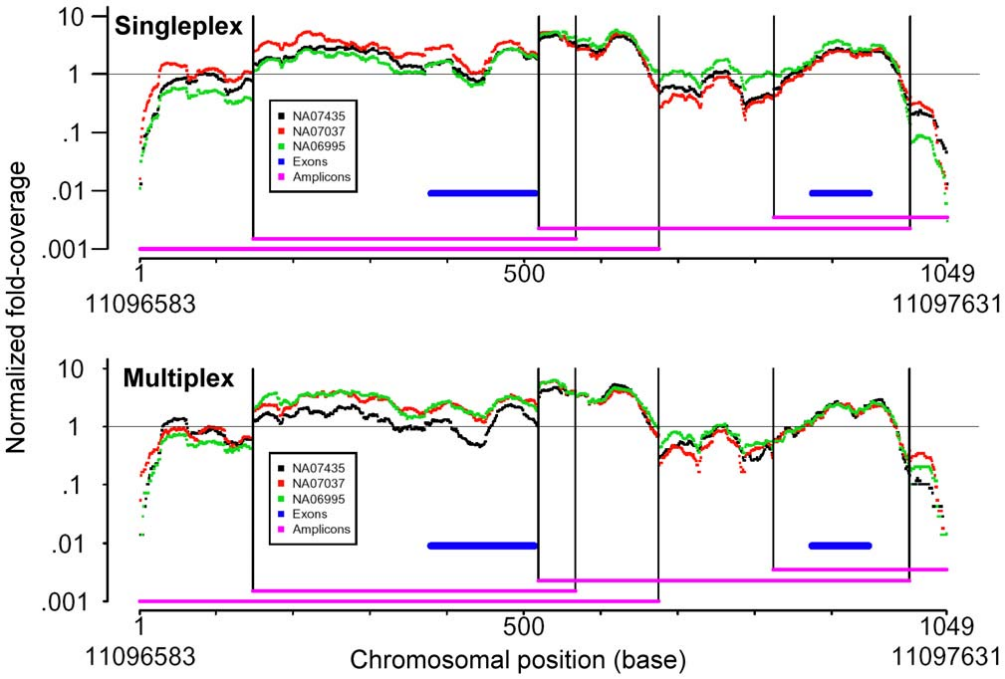
Comparison of targeted resequencing of independent samples. We show an example of a 1,049 base captured region, occurring between coordinates 11096583 and 11097631 of chromosome 1. The fold-coverage from the three samples has been normalized by taking the ratio of fold-coverage at each position to the median depth for the sample, and then taking the log_10_ of that ratio. Purple lines indicate a capture oligonucleotide's target. The exons are indicated by the blue lines. Vertical lines, extending from the beginning and end of each captured amplicon, show that the discontinuities in depth are associated with the ends of captured targets.

In both the single and the multiplexed cases, the median normalized log FC at each base position is superimposable for all three family members over the entire length of a typical targeted region (Fig. 3). Sharp transitions in FC occur where the capture oligonucleotides begin and end (*e.g.* around positions 150, 550 and 1,000). This is expected as the FC represents the sum of all the oligonucleotides capturing a given region. The three FC curves are highly correlated in both panels.

We analyzed the reproducibility of FC across three datasets derived from three independent 10-gene capture assays of three CEU individuals. Sequencing was conducted both as simplex with a single capture sample per lane and multiplex with three samples sequenced in a single lane. After alignment, total sequencing coverage was median normalized. Median and average values were calculated for all six samples (Text S1). Correlations between simplex datasets are shaded in green and correlations between multiplex dataset are shaded in purple. The Pearson's correlation between all pair wise combinations of the six datasets was calculated. The average correlation between multiplexed sequenced datasets is slightly higher (0.96) than between simplex lanes (0.94) for these three samples.

### SNV analysis of targeted resequencing data

As an additional check of our capture assay performance, we compared our SNV calls from the targeted resequencing data to the reported Hapmap data for the samples we sequenced (Table 4). For capture assay 1, we have perfect concordance with Hapmap genotype data in CEU individuals NA07037 and NA06995. In NA07435, we have a single false negative heterozygote. In capture assays 2 and 3, the SNV discovery sensitivities range between 92.2% and 98.5% while the specificities range from 98.5% to 99.7%. The target size and assay yields are comparable between the two experiments. In addition to the Hapmap SNP genotypes, we detected other heterozygote and homozygote variants in the CEU individuals in the 10-gene capture assay. The majority of these variants are reported in dbSNP (Table 4). All novel variants are listed in Text S1. A similar trend was also observed for the larger capture assays.

A complete genome sequence of NA18507, a Yoruban individual, was published by Bentley et al. [1]. We analyzed NA18507 with two separate replicates of capture assay 3, which covered 943 Kb, and we compared our results to the genotype calls reported from the full genome sequence. For replicate 1, we detect 582 variants in common with Bentley et al., 89% of which are in dbSNP, the remainder being novel variants. To assess additional variants unique to our sequence data we adopted the exact same procedure as reported by Bentley et al., which involves filtering short read misaligned repetitive sequences [1]. This led us to call 28 additional SNVs not called by Bentley. Among these 28 variants, 18 are present in dbSNP (65%). We conducted Sanger sequencing for additional confirmation on the remaining 10 possible variants. We confirmed that four of these were heterozygotes by Sanger sequencing and six were not. Likewise, replicate 2 was nearly identical in terms of the SNVs we identified.

### Insertion and deletion analysis

Detection of insertions and deletions (indels) from single short read sequences lacking a mate pair is extremely challenging. Further complicating indel detection, our fragmentation method involves random concatenation of the captured material followed by sequencing library preparation. While this method provides adequate FC over the targets, it essentially precludes the use of mate pairs for identifying indels since mate pairs from a given Illumina sequence cluster have a high probability of coming from different captured amplicons. As a solution for identifying indels from single reads less than 50 bases, we aligned the sequence data with the Illumina alignment program, Eland v2 (Text S1). This recent version of Eland does simple indel detection by default using a gapping procedure, and could only be implemented on the later sequencing runs. Eland v2 introduces a gap when both the following conditions are met: i) the gap reduces the number of mismatches by 5 compared to the corresponding non-gapped alignment and ii) the ratio of mismatches given no gap to mismatches given a gap is at least 3 to 1. Therefore, the size of a detected indel is closely tied to the length of the sequence read and our short length handicaps the detection. Using a high specificity set of criteria on the 106-gene capture assay, we called ten indels from NA18507 among which six are listed in dbSNP and all are also listed in Bentley et al [1]. We note that before filtering to our desired level of stringency, our alignment had reported 51 of the 87 indels noted by Bentley et al. in the same overlapping target region. For NA07037 with capture assay 2, we found five indels all of which are listed in dbSNP129. In the case of our matched normal - tumor colorectal cancer pair, no somatic indels specific to the tumor were identified.

### Mutation discovery from analysis of a matched normal tumor pair

We developed another analysis procedure for determining somatic mutations from a matched normal colorectal adenocarcinoma pair using capture assay 3. Here, we determine the difference in the percentage of FC represented by the variant base between the two sequenced samples. We determine the standard error (SE) of the difference between the matched samples and calculated a 95% confidence interval. The procedure is applied independently to data from the forward and the reverse reads, as described for the SNV discovery, and we only consider the tumor specific variants where there is double strand confirmation. In the analysis of a colorectal matched pair (2950N, 2951T) we identified multiple cancer mutations (Table 5). These included mutations in *KRAS* and *APC*, two somatic genetic changes frequently observed in colon cancer. Of particular interest is a *KRAS* gene mutation occurring in codon 12 (G12D) which is a hot spot mutation commonly identified in colorectal cancer [20]. Approximately 90% of the activating mutations are found in this particular codon, which represents a highly specific mutation for colorectal carcinoma [21]. Several groups have recently validated that *KRAS* mutations are a negative predictor of colorectal carcinoma response to monoclonal antibodies (e.g. panitumimab and cextuximab) targeting epidermal growth factor receptor (EGFR) [22], [23].

**Table 5:** Tumor specific mutations in matched normal tumor pair.

Gene	Chr	Gene location	Genomic position	cDNA position	*Coding change*	*Colon cancer (2951T)*	Normal colon (2950N)
						***Fraction of sequence reads with the mutation***	
*TNNI3K*	1	Exon 20	g.ch:1:74677879G>A	c.2047G>A	*none*	31.7%	0.7%
*APC*	5	Exon 15	g.ch:5:112201816C>T	c.2646C>T	*none*	36.4%	1.9%
*BAI3*	6	Intron 14–15	g.ch:6:69842382G>A	NA	*NA*	32.8%	0.3%
*BRAF*	7	Intron 17–18	g.ch:7:140081065A>G	IVS18+25A>G	*NA*	25.8%	0.0%
*KRAS*	12	Exon 1	g.ch:12:25289551C>T	c.226C>T	*G12D*	33.3%	0.9%
*NAV3*	12	Intron 9–10	g.ch:12:76939845C>A	NA	*NA*	25.9%	5.0%

## Discussion

We have successfully developed an integrated targeted resequencing approach using selective genomic circularization. Our capture approach is geared towards resequencing gene subsets smaller than the scale of exome sequencing but larger than can be done efficiently with simplex PCR. Our method is most appropriate for applications where: i) low amounts of input DNA are available, in the range of tens of nanograms, ii) the target size is on the order of 1 Mb and potentially higher, iii) the number of samples assayed exceeds 100 and iv) high sequencing coverage is needed for improved sensitivity. We also developed the Human OligoExome resource (http://oligoexome.stanford.edu) that enables researchers to download capture oligonucleotide sequences and create their own customized capture assays. While exome sequencing has become commonly available, there are numerous applications and studies which oftentimes only require smaller scale of targeted resequencing such as the clinical analysis of Mendelian disorders showing genetic heterogeneity, the confirmation of mutations in newly discovered cancer genes from genome surveys and deep resequencing of loci identified by genome wide association studies.

In this initial application, we demonstrate that we can assess up to one Mb with over two thousand exons derived from *in silico* sequences for capture oligonucleotides found in the Human OligoExome resource. Capture success, as defined by a minimum level of ten-fold-coverage for a given target, was generally greater than 85%. We applied these custom-designed capture assays on both normal diploid genomic DNA and cancer samples. We believe that the capture size can be increased significantly, and are working to expand the assay capacity.

Our approach has low genomic DNA requirements; specifically, we tested a minimum starting amount less than 100 ng. To determine the performance reproducibility with low DNA template amounts, we assessed replicate NA18507 samples using 50 and 80 ng and discovered that the sensitivity and specificity of SNV discovery were essentially the same. This low DNA requirement makes our capture assay particularly useful for sequencing clinical samples where cellular quantities are limited, such as small biopsy samples and aspirates used in tissue diagnosis of cancer. For many applications, the low starting amount of genomic DNA template eliminates the need for whole genome amplification. The low genomic DNA requirements of our method is in contrast with the higher amounts required for commercially available exome capture methods such as SeqCap from Nimblegen [11] or SureSelect from Agilent [12], both of which require starting microgram amounts of genomic DNA.

We assessed the on-target specificity of capture of our assays and found it to be high. In capture assay 3, only 4.6% of the sequences appear to be derived from non-targeted regions of the human genome. On-target specificity is mediated by several factors, including the requirements for both capture arms to anneal to their complementary genomic target sequences. The capture arm adjacent to the restriction site must precisely anneal because the ligation of the vector oligonucleotide to the genomic DNA requires a precise junction for the ligase reaction to be completed. We demonstrated that the addition of uracils to the capture and vector oligonucleotides is extremely useful in eliminating excess oligonucleotides by means of a uracil-deglycosylase after the genomic circularization.

Our assay's capture efficiency can be adjusted based on individual oligonucleotide concentration changes that are readily handled by standard laboratory robotics. We can alter variance among captured targets by simply diluting or increasing the concentration of a specific oligonucleotide. Given that the capture assay requires a low concentration of each individual oligonucleotide, typically 5 to 500 pM in 20 ul reaction volumes, a traditional oligonucleotide synthesis typically yielding 10 nM of material has the potential to provide assays for a large number of samples (e.g. up to 10^6^ assays for oligonucleotides used at 500 pM). The low oligonucleotide requirement significantly reduces the overall cost of the capture assay and as such, reduces the cost of targeted resequencing in large population genetic studies that could involve hundreds or thousands of samples. We also conducted a cost analysis of the method and using an assumption of an analysis of 1,000 samples our overall (enzymes, disposables and oligonucleotides) cost per sample was approximately $75 (Text S1).

Given that the capture occurs in solution, we believe that automating the assay for increased sample throughput should prove to be straightforward. We also demonstrated that indexing of the different samples is a practical solution for increasing the number of samples per sequencing lane. The success of indexing shows the practicality of this approach in targeted resequencing of clinical populations. Reproducibility of the assay was excellent as demonstrated by comparing simplex to multiplexed sequencing samples.

We used an Illumina Genome Analyzer to conduct our resequencing and developed a SNV discovery method with high levels of statistical confidence based on single short read sequences less than 50 bases in length. The application of forward and reverse strand confirmation and repeat masking facilitated highly accurate SNV discovery. Our sensitivity improved dramatically during the course of development because of radical improvements made in the capture assay and the remarkable increases in sequencer output. We anticipate that using longer reads should further improve our SNV discovery specificity. Longer reads will also facilitate indel detection, which was severely hampered by the single short reads that we used.

## Materials and Methods

### Genomic DNA samples

Genomic DNA for NA07037, NA06995, NA07435 and NA18507 was obtained from the Coriell Institute for Medical Research (Camden, NJ). In the case of matched normal and primary tumor pairs, genomic DNA was extracted from a matched normal tumor colorectal cancer pair (2950/2951) by using the DNAeasy Tissue Kit (Qiagen) following the manufacturer's protocols. All patient material was obtained with informed consent from the Stanford Cancer Center and the study was previously approved by the institutional review board at Stanford University School of Medicine.

### Capture oligonucleotide design

We utilized a novel bioinformatic design process, referred to as TargetedOligoDesign, which enabled us to design targeting oligonucleotides for the entire CCDS exon definition set. This represents a significant advance over our previous design process [24]. The Stanford Human OligoExome dataset and the capture assays described here used CCDS build release 20080902 (ftp://ftp.ncbi.nlm.nih.gov/pub/CCDS/archive/Hs36.3/), human genome build NCBI 36.3 (ftp://ftp.ncbi.nlm.nih.gov/genomes/H_sapiens/) and dbSNP Build ID 129 (http://www.ncbi.nlm.nih.gov/SNP/) as the polymorphism reference data set. Briefly, TargetedOligoDesign uses as its inputs the genomic coordinates' ROI, which encompasses exon boundaries, adjacent intronic sequence and known SNPs. It takes into consideration the sequence of the target region, and the recognition site sequence of the restriction enzyme being tested. We used four restriction enzymes (*Mse*I, *Bfa*I, *Sau*IIIA or *Cvi*QI), all of which recognize 4 bp sites. The TargetedOligoDesign program takes into account three factors. First, at least one end of the probe oligonucleotide is positioned adjacent to a restriction site in the target region, in order to mediate circularization. Second, the entire targeted region must be less than 800 bases in length in order to facilitate efficient circularization and amplification of the ROI. Third, the oligonucleotide's capture arms must not hybridize to known SNP positions. Initially, TargetedOligoDesign designs a probe to capture an entire ROI if the amplicon is less than 800 bases in length. The oligonucleotides' capture arms are designed to hybridize directly 3′ of the 5′ restriction site and 5′ of the 3′ restriction site in the target region. In cases where the resulting amplicon is greater than 800 bases in length, one capture flank is designed to hybridize to the boundary of the ROI, while the other target specific region continues to hybridize directly to the inside of the restriction site, creating a flap of external sequence that can later be cleaved using the exonuclease activity of Taq polymerase (Text S1). The strand targeted by the capture oligonucleotide is by the structure of the circularized intermediate. When one or more restriction sites internal to the ROI are present, TargetedOligoDesign will design multiple probes that tile the region.

### Annotation of oligonucleotides with quality control features

We analyzed the sequences of all oligonucleotides' capture arms in order to identify potential issues that might cause failures or off-target capture. Four different quality control factors were reviewed for each oligonucleotide and any oligonucleotides that failed to pass them are annotated with flags. First, if one or the other of the 20 base capture arms from a single oligonucleotide is present more than once in the human genome sequence, the oligonucleotide is designated with a “W” flag. Second, if both capture arms are duplicated in the human genome and duplicate capture arms for a given oligonucleotide are within 1 Kb distance of one another and in phase, the oligonucleotide is designated with a paralog “P” flag. Third, if the outer 14 bases of either capture arm perfectly match an Alu consensus the oligonucleotide is tagged with an “A” flag. Fourth, if the same outer 14 bases match any other repeated sequence (e.g. not an Alu) present in a consensus repeat list derived from Repbase, a data set of human genome repeats [25], [26], that oligonucleotide is designated with a “R” flag. The comparison against the human genome was conducted with SeqMap [27] run against each human chromosome (e.g. seqmap 1 input.fa hs_ref_chr1.fa output_vs_1/output_statistics), which identifies the number of perfect matches and single mismatch alignments separately. The comparisons against consensus repeat sequences used in the assignment of the A and R flags are performed using simple string comparison functions in Matlab.

### Stanford Human OligoExome database

The Stanford Human OligoExome resource (oligoexome.stanford.edu) runs on a 2×2.27 GHz Quad Core Intel Xeon E5520 server, with 24 GB memory, and Ubuntu 9.10 operating system. The web application is implemented in Ruby on Rails 2.3.8, running under Passenger 2.2.15. The underlying database is MySQL 5.0.42 community edition, which is hosted on a separate database server. Query and data download is via any current web browser. Recommended browsers and versions are: Internet Explorer 7.0+, Firefox 3.0+, Safari 5.0+, Chrome (any version).

### Capture assay method

After creation of an *in silico* data set of 80-mer oligonucleotide designs for the exons within CCDS, we selected those which covered the sets of genes as previously described. All oligonucleotides were synthesized at the Stanford Genome Technology Center and pooled based on those oligonucleotides specific to each restriction enzyme. This resulted in three separate pools for each capture assay. The capture oligonucleotides are described in Text S1. The universal vector sequence and primers are described in Text S1. The 10-gene capture assay was performed as described previously [14]. In the case of capture oligonucleotides specific for *Sau*3AI, *Dpn*II, a *Sau*3AI isoschizomer that recognizes the same palindromic sequence, was the actual enzyme used for restriction digests. For capture assays 2 and 3, we further optimized the protocol to reduce the input genomic DNA requirement and increase the total length of the targeted genomic regions. Briefly, a total of anywhere from 50 to 250 ng human genomic DNA was digested to completion with 3 to 5 units of *Mse*I, *Bfa*I, *Dpn*II or *Cvi*QI restriction enzyme (New England BioLabs). Subsequently, one third of each digestion was combined with 2.5 unit each of Ampligase (Epicentre Biotechnologies) and Taq polymerase plus 50 pM each of the capture oligonucleotide pool and the vector oligonucleotide at equimolar concentration with the capture oligonucleotide pool. The reactions were first denatured at 95°C for 5 minutes and then subjected to 10–15 cycles at 95°C for 1 minute, 60°C for 45 minutes, and 72°C for 15 minutes. Under these conditions, the captured genomic regions formed partially double-stranded circles via oligonucleotide-mediated nick ligation. Uracil excision enzymes (Epicentre Biotechnologies), at 1 unit per reaction, were used to linearize the circles and degrade excess targeting and vector oligonucleotides. After a brief purification using the Spin-20 columns (Princeton Separations), the captured DNA pool was amplified by PCR (98°C for 30 seconds followed by 36–37 cycles at 98°C for 10 seconds, 65°C for 30 seconds, and 73°C for 30 seconds) using Phusion Hot Start High-Fidelity DNA polymerase (New England BioLabs) and non-target specific common primers that are homologous to the vector oligonucleotide.

After purification using the Fermentas PCR Purification kit, 0.5–1 µg PCR products per sequencing library were ligated to each other using T4 DNA ligase (New England BioLabs). For capture assays 2 and 3, the concatenated amplicon DNA was fragmented using the Bioruptor (Diagenode), a probe-free sonication device. Capture assay 1 assay was fragmented with enzymatic DNAseI treatment. We used a 1∶400 dilution of DNAseI (2000 U/ml) (New England Biolabs) in 50 mM Tris pH 7.5, 10 mM MnCl_2_, and 50 µg BSA. A volume of 100 µl of the diluted DNAse solution was added to the concatenated material and incubated at 37°C for 10 minutes. After 10 minutes, the reactions were terminated by adding 6 µl of 0.5 M EDTA on ice, followed by a 10 minute 75°C inactivation. Subsequent sequencing library preparation was essentially as described in [28] with minor modifications. For the “A” tailing step prior to ligation to the adapters, we used Taq polymerase for improved efficiency and shorter reaction time [29]. Size selection of the sequencing libraries in the range of 200–300 bp was accomplished by using the 2% SizeSelect E-Gel (Invitrogen).

### Illumina sequencing and simplex PCR – Sanger sequencing validation

Resequencing was conducted with an Illumina Genome Analyzer I and II. The samples were sequenced according to the manufacturer's specifications. Images were collected and after the run, image analysis, base calling and error estimation were performed using Illumina sequencing software (version 2.2.195 through version 2.6.26) and analysis pipeline software (1.01 through 1.6). For capture assay 1, samples were sequenced in 36 single-read cycles, analyzed with pipeline 1.01 and aligned using MAQ (7.1) with default parameters –n 2, -e 70. For capture assay 2, samples were sequenced in 42 single-read cycles, analyzed using Illumina RTA 1.5.35 and aligned with Eland, from Illumina software analysis pipeline version 1.5. For capture assay 3, samples were sequenced in 42 paired-end cycles, analyzed using Illumina RTA 1.6.32 and Eland v2, pipeline version 1.6. A PhiX control lane was used for all image analysis. Alignments used default parameters. Double-stranded Sanger sequencing on amplified exons was carried out on a number of variants as confirmation. Standard PCR and Sanger sequencing was performed similarly to as presented in Liu *et al.* [30]. For the validation of novel SNVs, the gene specific portion of each primer pair used in Sanger sequencing was derived from a previously published list of primers [8]. Each forward and reverse primer was tailed at its 5′end with the M13 universal forward and reverse sequences respectively.

### Capture assay adjustment

To evaluate the performance of individual targeting oligonucleotides, we assayed normal genomic DNA samples (NA07037 and NA06995) and sequenced the three enzyme-based sub-pools on separate lanes. For each capture oligonucleotide, using the sequence alignment data we calculated the average fold-coverage per base achieved over the entire length of the resulting amplicon. For variant discovery purposes, we assumed that an average FC ranging from 100 to 1,000 was an optimal performance range with FC's less than 100 or greater than 1,000 being too extreme for efficient accurate variant discovery and use of sequence capacity, respectively. We used a Biomek robot (Beckman-Coulter) to dispense the oligonucleotides for the normalized reaction with concentration for each oligonucleotide listed in Text S1.

### Repeat masking

Given the issues with aligning short sequences to repetitive regions and the potential for resulting false SNVs, we developed a repeat masking procedure tailored specifically to short reads. With this capture assay system, repeats can be generated either i) by repetitive elements in the intronic regions adjacent to the exons or ii) within the exons either by repetitive motifs or by paralogs or pseudogenes which have sequence similarity to the primary targeted exon. Using the target reference sequence, we created a series of 14 base sequences via a one base increment tiling across the entire genomic target interval. We determined whether these 14 base tiles mapped to a list of consensus Repbase repetitive elements derived from the human genome (http://www.girinst.org/repbase) [25], [26]. Using sequence data generated from Hapmap individuals, we discovered that tile sequences shorter than 14 bases eliminated true SNVs while tile sequences longer than 14 bases produced a substantially higher number of false positives. If a specific tile has a perfect match to a sequence of a repetitive element, each of the coordinates within the tile receives a score of 1. Additional perfect matches of overlapping tiles are added to each base coordinate to create a summary repeat masking score for every genomic coordinate within the target. To address the issue of repeats not annotated by Repbase, we also determined whether short sequences from a specific target region-of-interest were represented multiple times in the human genome, as may occur in pseudogenes. For this additional repeat masking procedure, we use the same reference sequence and increase the tile size to 36 bases given that we are now comparing to a much larger sequence. This procedure results in a quantitative genome repeat masking score for each base position. The comparison against the human genome was conducted by running SeqMap [27] against each human chromosome (e.g. seqmap 1 input.fa hs_ref_chr1.fa output_vs_1/output_statistics), which identifies the number of perfect matches and single mismatch alignments separately. The results of both repeat masking procedures were combined as a logical “OR” for our analysis. Any genomic reference coordinate which is not zero in either repeat masking score or genome masking score was eliminated from subsequent SNV discovery.

### Detection of SNVs

For SNV detection of our targeted resequencing data, there are multiple methods available. We tested four methods and compared the results to determine their performance with our capture sequence data against Hapmap genotypes from the capture region. These include MAQ [31], the SAMtools implementation of the SOAPsnp model [32], the Genome Analysis Tool Kit (GATK) [33] and our own SNV detection method which was adapted for this targeting assay metrics. Comparison to the Hapmap genotype results indicated that the accuracy of our calling algorithm is 98%. This accuracy is similar to that of MAQ (97%), GATK (96%) and SOAP (97%). Given the comparable performance among all four SNV callers and we opted to use our method as described below.

For MAQ, each of the paired-end reads was aligned separately using default values: maq map (-n 2 -e 70); they were combined with the merging function: maq mapmerge. The consensus was generated using default maq assemble on the combined map file to produce a cns file, and then maq cns2view was run on the cns file in order to produce a list of calls at all positions. For other methods, the first step taken was aligning the concatenated list of paired-ends reads (i.e. each read treated as a single end) using BWA, a Burrows-Wheeler aligner. The command used was bwa samse, with default parameters. The two methods which used this index were SAMtools and GATK. SAMtools (http://samtools.sourceforge.net) was run using the SOAPsnp model developed for the aligner suite SOAP (http://soap.genomics.org.cn) as a SNP caller. The steps taken were: samtools pileup -avcf, to get variants only (-v) based on consensus (-c). Requiring calls at every position led to a much higher number of errors. Therefore we required a depth of at least 10 and no limitation on the maximum fold coverage with the following: samtools.pl varFilter -d 10 -D 10000000. The results were then filtered to remove calls with quality score below 20. GATK is a set of tools developed at the Broad Institute (ftp://ftp.broadinstitute.org/pub/gsa/GenomeAnalysisTK/), which includes SNV caller. As a first step, the Unified Genotype element of GATK was used with default quality score (-stand_call_conf 50.0) and specifying the platform as solexa (–platform SOLEXA) in order to call SNPs: java -jar GenomeAnalysisTK.jar -I input.bam -R ref.fa -T UnifiedGenotyper -varout GATKsnpCalls.vcf -stand_call_conf 50.0 -S SILENT –platform SOLEXA. Putative SNPs were then filtered according to metrics recommended on the GATK wiki (http://www.broadinstitute.org/gsa/wiki/index.php/): java -jar GenomeAnalysisTK.jar -T VariantFiltration -R ref.fa -o snpCalls.filtered.vcf -B variant,VCF,GATKsnpCalls.vcf –clusterWindowSize 10 –filterExpression “AB>0.75 && DP>40 || SB>−0.10” –filterName “StandardFilters” –filterExpression “MQ0> = 4 && ((MQ0/(1.0 * DP))>0.1)” –filterName “HARD_TO_VALIDATE”. In particular, –clusterWindowSize 10 flags cases where there are 3 or more SNPs within 10b of one another.

For our own SNV procedure, our algorithm takes into account i) FC at the variant base call, ii) appearance of the variant base call on complementary forward and reverse strand reads (hereafter called “double-strand confirmation”) and iii) repeat masking of repetitive sequences, which can introduce false positive variants. In our SNV analysis process, for each coordinate position in the reference sequence, the number of calls of each base is first counted, both matches and mismatches. This becomes a mapped sequence matrix consisting of the chromosome coordinate of the reference and the fold-coverage of each base call (i.e. the number of A, C, G and T base calls). We developed an analysis pipeline for variant detection using this mapped sequence matrix as input. Let 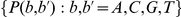 be the matrix of probabilities of observing 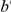 when the true base is 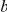. Let 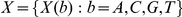 be the fold-coverage for a given base at a specific coordinate position. Under the null model where the reference coordinate is homozygous base 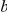, 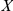 should be multinomial with probability distribution 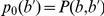 for 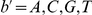. Under the alternative model where the position is heterozygous with 50% base call 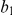 and 50% base call 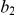, then 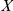 should be multinomial with a mixture distribution
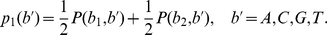
In reality, we do not know what the true underlying base is, but the most frequently observed base at the position (*i.e.* the base with the highest fold-coverage (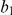) at any given position) is typically the true base call. If the position is heterozygous, then the two bases with the highest (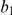) and second highest (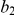) fold-coverage usually represent the two alleles. The log likelihood ratio statistic for testing the alternative model against the null model is
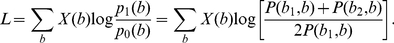
(1)Using the forward and reverse complementary reads separately, we compute the likelihood ratio statistic 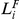, 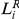 for each position 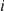. In the first pass, we propose as candidates all potential SNV positions where *both* the forward and reverse strand likelihood ratio statistics are higher than a certain threshold:
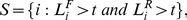
(2)where 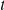 is a user-chosen threshold. Requiring both the forward and reverse strand confirmation of a variant filters out many errors that are specific to the sequence processing. Subsequently, all positions in 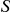 that lie in repeated regions of the genome are repeat masked.

The probability matrix 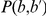 can be computed by tabulating the fraction of times 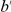 is read, when the most frequent base is 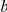, over all positions in the sequenced region. Since we assume only a very small fraction of total positions are heterozygotes, the true heterozygotes contribute little to the fraction. For greater accuracy, the process can be iterated; that is, after excluding the heterozygotes identified using 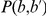 computed from all positions, 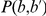 can be recomputed, and used to re-compute the likelihood scores.

### Indel detection

To determine the presence of indel variants from single sequence reads of less than 50 bases, we aligned the sequence data with Eland2, an alignment application available with version 1.6 of the Illumina Genome Analyzer software. Based on our capture assay of NA18507, which had complete genome sequencing data available, we developed a procedure to eliminate false positive indels. First, we required sequence data from at least two breakpoints in both forward and reverse alignments, where breakpoint is the first position of a read. Second, the indel sequence had to have FC at the 5′ position greater than 50. Third, we required that a sequence containing the indel fail to align against the genome with less than 3 mismatches. This minimizes the risk that an off-target captured sequence would be falsely called as an indel. Eland2's indel detection is heavily dependent on the read lengths of the sequences involved, however, so the relatively short sequencing lengths of the assays in this paper preclude extensive indel discovery.

### Detection of mutations in matched normal tumor sample

For identifying somatic mutations from matched normal and tumor samples, we required a more sensitive test to detect somatic and cancer-specific SNVs. We developed a method based on *t*-test statistics and confidence intervals for targeted resequencing of matched tumor normal pairs using double-strand confirmation. For any fixed position, let 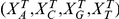 be the base counts in tumor, and 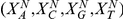 be the base counts in the matched normal. Let 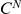 and 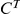 denote respectively the fold-coverage in normal and tumor. Define
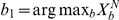
to be the base with the highest count occurring in the normal, and
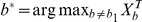
to be the base (other than 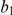) with the highest count in the tumor. Let
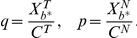
Then, define
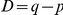

*D* is the difference in count of the base 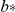 between the tumor and the normal. If this position were a homozygote for the same base in both normal and tumor, then *D* would be small in absolute value. If this position were a homozygote in normal that has turned into heterozygote in the tumor, then *D* would be a large positive fraction. This method does not detect heterozygote to homozygote shifts as in regions of loss of heterozygosity, which we identify by first isolating the heterozygous positions in the normal and then computing the difference in the counts between the two alleles in the tumor.

The confidence interval for *D* factors in the coverage in both the normal and tumor samples. If the coverage is high in both samples, then the confidence interval is narrow. If *either* sample has low coverage, then the standard error in *D* is large, leading to a wide confidence interval. The equation for the standard error of *D* is
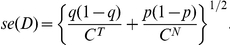
With sufficient depth, *D* is approximately *t* distributed. The 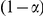 confidence interval for *D* can then be computed based on the quantiles of the appropriate Student-*t* distribution. With the confidence intervals computed, the filtering rule is based on the 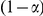 confidence interval and a minimum acceptable value 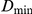 for the absolute change 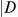. We report positions where 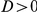 and the lower confidence interval is above 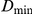, or 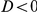 and the upper confidence interval is below 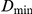. In this study we have used 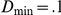 and 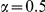.

## Supporting Information

Figure S1 **The selective genomic circularization process.** Genomic DNA is digested with one of several possible restriction enzymes. The restriction digest is mixed with a pool of targeting oligonucleotides and a single 40 base oligonucleotide vector. Each targeting oligonucleotide has two 20 base capture arms complementary to genomic DNA. One of the capture arms is positioned exactly at the end of the restriction fragment the other arm may be placed internally to the restriction fragment. The 5′endonuclease activity of TaqI polymerase degrades the 5′ extension if present and ligase circularizes the intermediate. The UDG reaction degrades the targeting oligonucleotide and linearizes the circle. Double stranded linear products are then generated by PCR using a pair of common primers.
(TIF)

Text S1 **Supporting tables and description of the cost assessment.**
(DOC)
