# Supplementary figures and images for: A Flexible Approach for Highly Multiplexed Candidate Gene Targeted Resequencing
Source: PLoS One. 2011 Jun 30;6(6):e21088. doi: 10.1371/journal.pone.0021088 (PMC3127857; doi:10.1371/journal.pone.0021088)

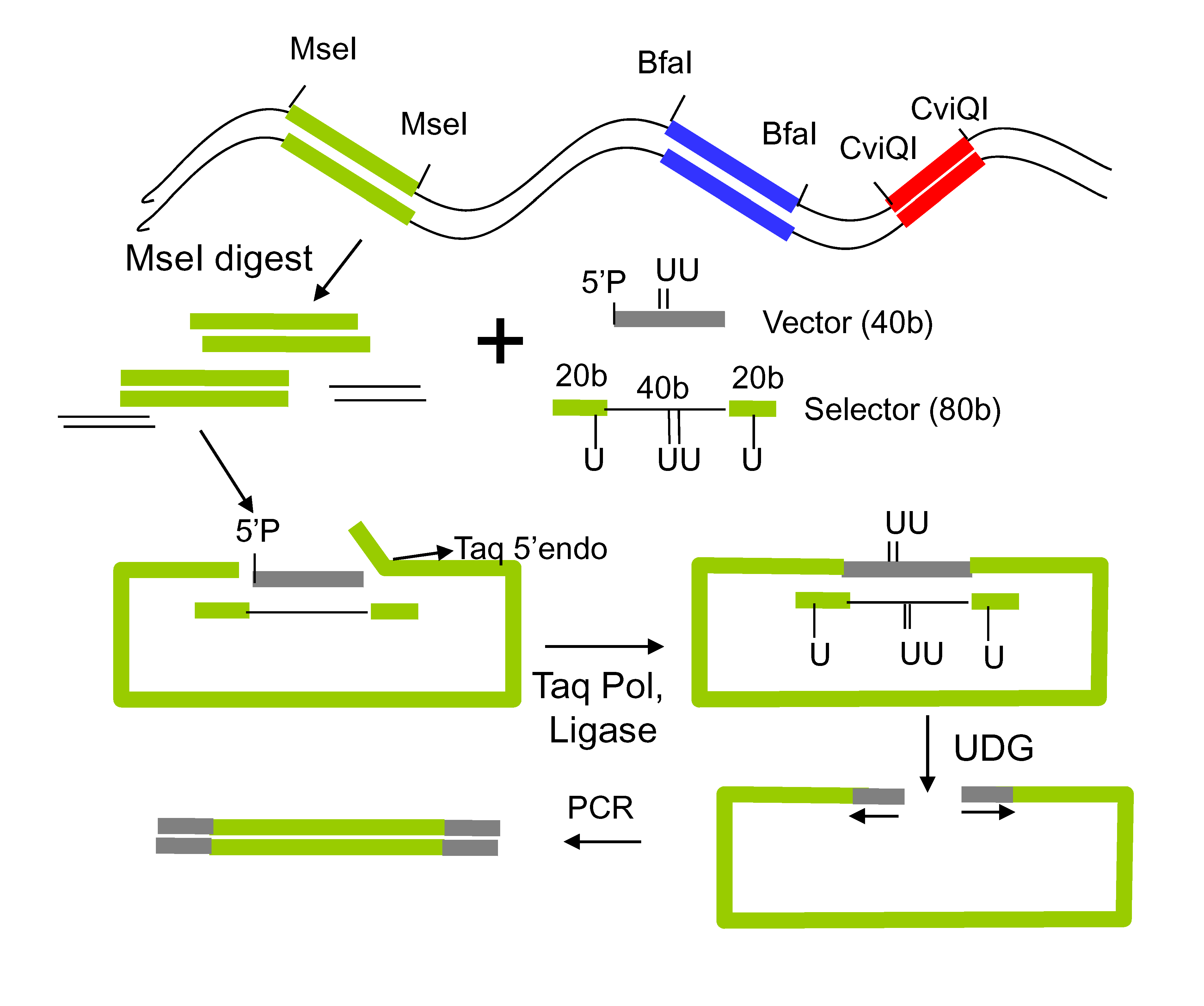

Supplement: Figure S1 — The selective genomic circularization process. Genomic DNA is digested with one of several possible restriction enzymes. The restriction digest is mixed with a pool of targeting oligonucleotides and a single 40 base oligonucleotide vector. Each targeting oligonucleotide has two 20 base capture arms complementary to genomic DNA. One of the capture arms is positioned exactly at the end of the restriction fragment the other arm may be placed internally to the restriction fragment. The 5′endonuclease activity of TaqI polymerase degrades the 5′ extension if present and ligase circularizes the intermediate. The UDG reaction degrades the targeting oligonucleotide and linearizes the circle. Double stranded linear products are then generated by PCR using a pair of common primers. (TIF) [file pone.0021088.s001.tif]
