# Supplementary material for: A Flexible Approach for Highly Multiplexed Candidate Gene Targeted Resequencing
Source: PLoS One. 2011 Jun 30;6(6):e21088. doi: 10.1371/journal.pone.0021088 (PMC3127857; doi:10.1371/journal.pone.0021088)
Supplement: Text S1 — Supporting tables and description of the cost assessment. (DOC) [file pone.0021088.s002.doc]

# Supporting Information

**Cost assessment**

Our capture technology is geared towards resequencing gene subsets substantially larger than can be done efficiently with simplex or multiplex PCR but reduced in scale compared to exome sequencing. Costs are described in Table S7. Given that a number of capture methods are available commercially, we assessed the potential utility and total cost of our capture approach including sequencing library reagent creation. Our cost analysis of reagents and disposables assumes the following:

1. The costs are based on overall price per vendor including sales tax and shipping.
2. We use three different restriction enzymes for our capture method (e.g. *Mse*I, *Bfa*I, *CviQ*I).
3. Batches of seven samples are processed in parallel in order to fill one Illumina flow cell (assuming one PhiX control lane) in an Illumina GAIIX instrument.
4. The overall target size is approximately 1Mb, which can be captured by approximately 5,000 oligonucleotides as we detail in the manuscript.
5. The cost per a uridine-containing 80-mer is $5 and yield is 10nM. Given the amount we require, this is an amount sufficient to perform up to 1 million reactions.
6. Oligonucleotide costs are amortized over 100, 1,000 or 10,000 samples

Given that the capture assay requires a low concentration of each individual oligonucleotide, typically 5 to 500 pM in 20 ul reaction volumes, a traditional oligonucleotide synthesis typically yielding 10 nM of material has the potential to provide assays for a large number of samples (e.g. up to 10^6^ assays for oligonucleotides used at 500 pM). For target sizes of 1Mb and sample sizes on the order of 1,000 or higher the total cost is dominated by the cost of reagents and disposables. With a sample size of 100, the total cost is just $301, most of which is accounted for by the oligonucleotides. Above 1,000 samples the cost of oligonucleotides becomes negligible. The cost per sample for exome enrichment of methods such as Nimblegen or SureSelect typically are greater than $1,000 per sample just for the capture assays and associated reagents. Therefore, for groups interested in sequencing gene sets smaller than exomes, there is a significant cost incurred for reagents. Array-based synthesis approaches can also be used to synthesize a large number of oligonucleotides. However, the setup cost can be as high as $50,000 and the number of samples that can be processed from the material recovered from one synthesis is unclear. Therefore, oligonucleotide costs will dominate the total cost per sample.

Sequencing costs are also proportional to target size for a given median fold coverage. We can routinely index 4 samples in a single lane of the Illumina GAIIx and achieve a median coverage greater than 100 over a 1 Mb target; whereas we need to combine 3 lanes of data for a single sample to achieve the same coverage when using exome capture. The recent increases in Illumina sequencing capacity due to the advent of the HiSeq2000 system make it even more cost effective to resequence candidate regions, genes and loci using our capture approach.

We believe that targeting in combination with indexing is optimal for the following applications:

1. Populations studies sequencing >100 individual samples such as may occur in candidate loci resequencing for novel variants in genome wide association studies (GWAS).
2. Moderate target size of 1-10Mb.
3. Applications where deep resequencing is required, such as validation of candidate variants identified by genome scale discovery techniques or analysis of mixed samples such as solid tumors.

**Supporting Tables**

**Table S1. Commercially available 4 base recognition Restriction Enzymes**

| Hpy*CH4V* | TGCA |
| --- | --- |
| Alu*I* | AGCT |
| Hae*III* | GGCC |
| NlaIV | GGNNCC |
| *CviQI* | GTAC |
| Bst*UI* | CGCG |
| Cac*8I* | GCNNGC |
| Mse*I* | TTAA |
| Taq*I* | TCGA |
| Sau*3AI* | GATC |
| Hha*I* | GCGC |
| Hpa*II* | CCGG |
| Fat*I* | CATG |
| Bfa*I* | CTAG |

**Table S2. Genes included in each of the capture assays**

| **10 genes (capture assay1)** |  |  |  |  |  |  |  |  |  |
| --- | --- | --- | --- | --- | --- | --- | --- | --- | --- |
| *AKT1* | *AKT2* | *APC* | *EGFR* | *FRAP1* | *KRAS* | *MARK3* | *SMAD4* | *TGFBR2* | *TP53* |
| **106 genes (capture assays 2 and 3)** |  |  |  |  |  |  |  |  |  |
| *ABCA7* | *ACVR1B* | *ACVR2A* | *APC* | *ARAF* | *ARFRP1* | *ATM* | ***ATP5A1*** | *BAI3* | *BRAF* |
| *BRCA2* | ***CABLES1*** | *CACNA2D1* | *CAMKV* | *CCND1* | *CCNE1* | *CDC42BPB* | *CDH1* | *CDH10* | *CDH20* |
| *CDKN2A* | *CHD5* | ***CSMD3*** | *CTNNA2* | *CTNNB1* | *CYFIP1* | *DACH2* | ***DCC*** | *DCLK3* | *DLG3* |
| ***EED*** | *EGFR* | ***EPHA3*** | *ERBB2* | *ERN2* | ***EZH2*** | *FBXW7* | *FGFR3* | *FHOD3* | *FLG2* |
| *FOXP4* | *FRAP1* | *GPR124* | *GPR133* | *GRIA3* | *HM13* | *HNF1A* | *IL2RG* | *KBTBD11* | *KIT* |
| *KLHDC4* | *KRAS* | *MAP2K4* | *MAP2K7* | *MEN1* | *MGC16169* | *MLH1* | *MLL3* | *MSH2* | *MSH6* |
| *MYC* | *MYH2* | ***NAV3*** | *NEK11* | *NF1* | *NF2* | *NRAS* | *OMA1* | *OR10R2* | *PCDH15* |
| *PCDH18* | *PDZRN3* | *PIK3CA* | *PPP1R3A* | *PRKAA2* | *PTEN* | *REM1* | *RET* | *RIPK1* | *ROR1* |
| *ROR2* | *SF3B1* | *SLC1A6* | *SMAD2* | *SMAD3* | *SMAD4* | *SMARCA4* | *SMO* | *SRC* | *ST6GAL2* |
| ***SUZ12*** | *TAF1* | *TAF1L* | ***TCF4*** | *TGFBR2* | *TNNI3K* | *TNR* | *TP53* | *TPO* | *TRIO* |
| *TRRAP* | *TTK* | *TTN* | *TYK2* | *VHL* | *ZNF521* |  |  |  |  |

The ten genes in bold represent a subset unique to capture assay 3. The remainder are genes common to both capture assays 2 and 3

**Table S3. Analysis of factors affecting capture yield**

|  |  | **Capture assay 1** | |  | **Capture assay 2** | |
| --- | --- | --- | --- | --- | --- | --- |
|  |  | **360 Oligonucleotides** | |  | **2,341 Oligonucleotides** | |
| **Parameter** | **Variable** | **Number of oligos** | **Average fold-coverage per oligo target** |  | **Number of oligos** | **Average fold-coverage per oligo target** |
| **Amplicon length target (bases)** | <100 | 1 | 9,232 |  | 34 | 589 |
|  | <200 | 43 | 5,943 |  | 548 | 1,112 |
|  | 200-600 | 224 | 1,253 |  | 1,581 | 845 |
|  | 600-800 | 68 | 566 |  | 178 | 261 |
|  | >800 | 24 | 119 |  |  |  |
| **Targeting Arm %GC content** | > 75% | 33 | 842 |  | 66 | 304 |
|  | < 75% | 327 | 1,709 |  | 2,275 | 876 |
| **Presence of flap structure** | No flap | 124 | 1,824 |  | 835 | 1,229 |
|  | Flap < 1kb | 195 | 1,690 |  | 1,318 | 687 |
|  | Flap > 1kb | 41 | 756 |  | 188 | 429 |

**Table S4. Reproducibility of simplex and multiplex capture yield**.

|  | **NA07435 - simplex** | **NA07037 - simplex** | **NA06995 - simplex** | **NA07435 - multiplex** | **NA07037 - multiplex** | **NA06995 - multiplex** |
| --- | --- | --- | --- | --- | --- | --- |
| **NA07435 -– simplex** | 1.00 |  |  |  |  |  |
| **NA07037 -– simplex** | 0.94 | 1.00 |  |  |  |  |
| **NA06995 -– simplex** | 0.95 | 0.92 | 1.00 |  |  |  |
| **NA07435 -– multiplex** | 0.96 | 0.92 | 0.91 | 1.00 |  |  |
| **NA07037 -– multiplex** | 0.96 | 0.91 | 0.91 | 0.98 | 1.00 |  |
| **NA06995 -– multiplex** | 0.95 | 0.91 | 0.94 | 0.95 | 0.97 | 1.00 |
|  |  |  |  |  |  |  |
| **Median fold-coverage** | 151.00 | 380.00 | 348.00 | 69.00 | 55.00 | 69.00 |
| **Average fold-coverage** | 399.14 | 878.22 | 856.40 | 173.73 | 147.99 | 201.73 |

**Table S5: Novel SNVs**

Full table is available at [http://oligoexome.stanford.edu/](http://stanfordoligo.aptanacloud.com/).

**Table S6. Novel Indels**

| **Assay** | **Sample** | **Gene** | **Chr** | **Gene location** | **Genomic position** | **RefSNP ID** |
| --- | --- | --- | --- | --- | --- | --- |
| 106-gene | 2950 | *PRKAA2* | 1 | Intron 2-3 | g.ch:1:56912938_56912939insT | 11394634 |
|  |  | *PDZRN3* | 3 | Intron 3-2 | g.ch:3:73734312delA | 11343439 |
|  |  | *TRIO* | 5 | Intron 29-30 | g.ch:5:14451903delT | 34806703 |
|  |  | *EZH2* | 7 | Intron post 19 | g.ch:7:148135650delG | 3217095 |
|  |  | *EZH2* | 7 | Intron pre 2 | g.ch:7:148174627delA | 3214332 |
|  |  | *TRRAP* | 7 | Intron 38-39 | g.ch:7:98391676_98391677insC | 3214240 |
|  |  | *GPR124* | 8 | Intron 14-15 | g.ch:8:37815371_37815373delCTC | NA |
|  |  | *RET* | 10 | Intron 3-4 | g.ch:10:42920331delCC | 35906041 |
|  |  | *ATM* | 11 | pre exon23 | g.ch:11:107656918_107656919insA | 3218681 |
|  |  | *NF1* | 17 | Pre exon 14 | g.ch:17:26570114_26570115insT | NA |
| 96-gene | 7037 | *PDZRN3* | 3 | Intron 3-2 | g.ch:3:73734312delA | 11343439 |
|  |  | *BAI3* | 6 | Intron 15-16 | g.ch:6:69842768_69842769insA | 35445308 |
|  |  | *TRRAP* | 7 | Intron 11-12 | g.ch:7:98341644delG | 34508246 |
|  |  | *TRRAP* | 7 | Intron 38-39 | g.ch:7:98391676_98391677insC | 3214240 |
|  |  | *RET* | 10 | Intron 8-9 | g.ch:10:42927766delC | 34827976 |
| 106-gene | 18507 | *MSH6* | 2 | Intron post 7 | g.ch:2:47886379_47886382delCTAT | 2234731 |
|  |  | *PDZRN3* | 3 | Intron 3-2 | g.ch:3:73734312delA | 11343439 |
|  |  | *PDZRN3* | 3 | Intron 6-7 | g.ch:3:73522799_73522800insA | 35427155 |
|  |  | *EZH2* | 7 | Intron post 19 | g.ch:7:148135650delG | 3217095 |
|  |  | *EZH2* | 7 | Intron pre 2 | g.ch:7:148174627delA | 3214332 |
|  |  | *MLL3* | 7 | Intron 58-59 | g.ch:7:151465170_151465171insG | NA |
|  |  | *GPR124* | 8 | Intron 14-15 | g.ch:8:37815371_37815373delCTC | NA |
|  |  | *PCDH15* | 10 | Intron 30-31 | g.ch:10:55257406delA | NA |
|  |  | *RET* | 10 | Intron 8-9 | g.ch:10:42927766delC | 34827976 |
|  |  | *CYFIP1* | 15 | post Exon 14 | g.ch:15:20506749delC | NA |

**Table S7. Cost analysis of the selective circularization method**

| **Category** | **Type of reagent or disposable** | **Cost per sample ($)** |
| --- | --- | --- |
| **Enzymes** | Restriction enzymes | 1.83 |
|  | Ampligase | 2.25 |
|  | Taq polymerase | 1.66 |
|  | UDG | 4.65 |
|  | Phusion polymerase | 7.38 |
|  | DNA ligase | 2.70 |
|  | T4 polymerase | 2.90 |
|  | T4 PNK | 3.53 |
|  | Klenow | 0.66 |
|  | T4 DNA ligase, HC | 5.04 |
|  | dNTPS | 0.29 |
|  | dATP | 0.02 |
|  |  |  |
| **Disposables** | Spin-20 column | 7.35 |
|  | Fermentas PCR kit (2) | 4.64 |
|  | E-Gel SizeSelect Gels, 3 | 4.89 |
|  | PCR tubes | 0.45 |
|  | Agarose | 0.57 |
|  | Eppendorf tubes | 0.60 |
|  | TBE buffer | 0.57 |
|  |  |  |
| **Total reagents and disposables:** | | 51.98 |
|  |  |  |
| **Oligonucleotide costs** | |  |
|  | uridine containing 80-mer ($ per oligonucleotide) | 5.00 |
|  | total synthesis (5000 oligonucleotides) | 25,000.00 |
|  |  |  |
|  | Oligonucleotide cost amortized over | |
|  | 100 samples | 250 |
|  | 1,000 samples | 25 |
|  | 10,000 samples | 2.5 |
|  |  |  |
| **Total cost per sample (reagents, disposables and oligonucleotides),** | | |
| **amortized over :** | |  |
|  | 100 samples | 301.98 |
|  | 1,000 samples | 76.98 |
|  | 10,000 samples | 54.48 |

**Table S8: Capture Oligonucleotides**

**Full table is available on:** [http://oligoexome.stanford.edu/](http://stanfordoligo.aptanacloud.com/).

Sequence, genomic location and concentrations used for each oligonucleotide.

**Table S9. Primers for the universal vector.**

| **Vector oligonucleotide** | |
| --- | --- |
| vector40 | 5’ - CTCGACCGTTAGCAAAGCTUUAGCCTTGTACCGTTATCGT – 3’ |
|  |  |
| **Universal PCR primer pair used in capture reactions** | |
| forward_102 | 5’ - GGAGCTTTGCTAACGGTCGAG – 3’ |
| reverse_102 | 5’ - CCAGCCTTGTACCGTTATCGT – 3’ |
